# Pipeline variability: Friend or foe, depending on the goal: Comment on Germani et al. 2025

**DOI:** 10.1162/IMAG.a.1024

**Published:** 2025-11-26

**Authors:** Li-Bo Zhang, Tor D. Wager

**Affiliations:** Department of Psychological and Brain Sciences, Dartmouth College, Hanover, NH, United States

**Keywords:** pipeline variability, fMRI, mega-analysis

## Abstract

Germani et al. (2025, *Imaging Neuroscience*) argue that pipeline variability inflates false positive rates in between-group mega-analyses that “compare populations whose data were processed differently at the subject level.” While such variability is a confounder in this specific and rare type of analysis, it does not confound effects of interest in more common conventional mega-analyses, which test non-zero effects across studies. Instead, pipeline differences strengthen the generalizability of discoveries and protect against idiosyncratic pipeline-induced artifacts in conventional analyses. Even in between-group mega-analyses, it is context-dependent whether pipeline-induced differences are false positives. Recognizing these complexities will help understand when pipeline variability is advantageous and when it can cause problems.

Neuroimaging researchers routinely process their data with different pipelines, even when analyzing the same data to answer the same research questions (Botvinik-Nezer et al., 2020). Germani et al. (2025) assess the influence of such analytical variability in a specific type of neuroimaging mega-analysis that “compares populations whose data were processed differently at the subject level.” They contend that false positive rates can be inflated in these between-group mega-analyses when pipeline differences across studies are not accounted for. Although we agree with Germani et al. on the need for more attention to pipeline differences, we argue that (1) many mega-analyses are not vulnerable to the issue they raised, and (2) whether the term “false positives” applies to pipeline variability-related findings depends on the context, and it does not apply to many mega-analyses in practice.

To clarify the first point, we distinguish between conventional and between-group mega-analyses. Conventional mega-analyses, which are common in the literature, treat studies as replicates and identify findings (e.g., activation in brain areas) that generalize across studies (Zunhammer et al., 2018, 2021). A typical variant of this analysis tests whether the mean of a brain feature (e.g., activity level evoked by positive affect) deviates from zero across studies (Fig. 1A; Lindquist et al., 2016). Another variant compares two effects (e.g., positive affect vs. negative affect) at the within-study level, but still seeks to establish generalizability of these positive vs. negative differences across studies (Fig. 1B). These two variants usually perform a one-sample t-test or conceptually equivalent randomization test across studies to evaluate the effect of interest (Wager et al., 2009). The major difference between them is that, at the within-study level, variant 1 has only one group of subjects while variant 2 has two groups. By contrast, between-group mega-analyses, the type considered by Germani et al., test whether findings from one group of studies differ from those of another group of studies using the equivalent of a between-study two-sample t-test (Fig. 1C). For example, these analyses would compare a group of studies on positive affect with another group of studies on negative affect. As Germani et al. acknowledge, this type of mega-analysis is “not common practice.”

**Fig. 1.**
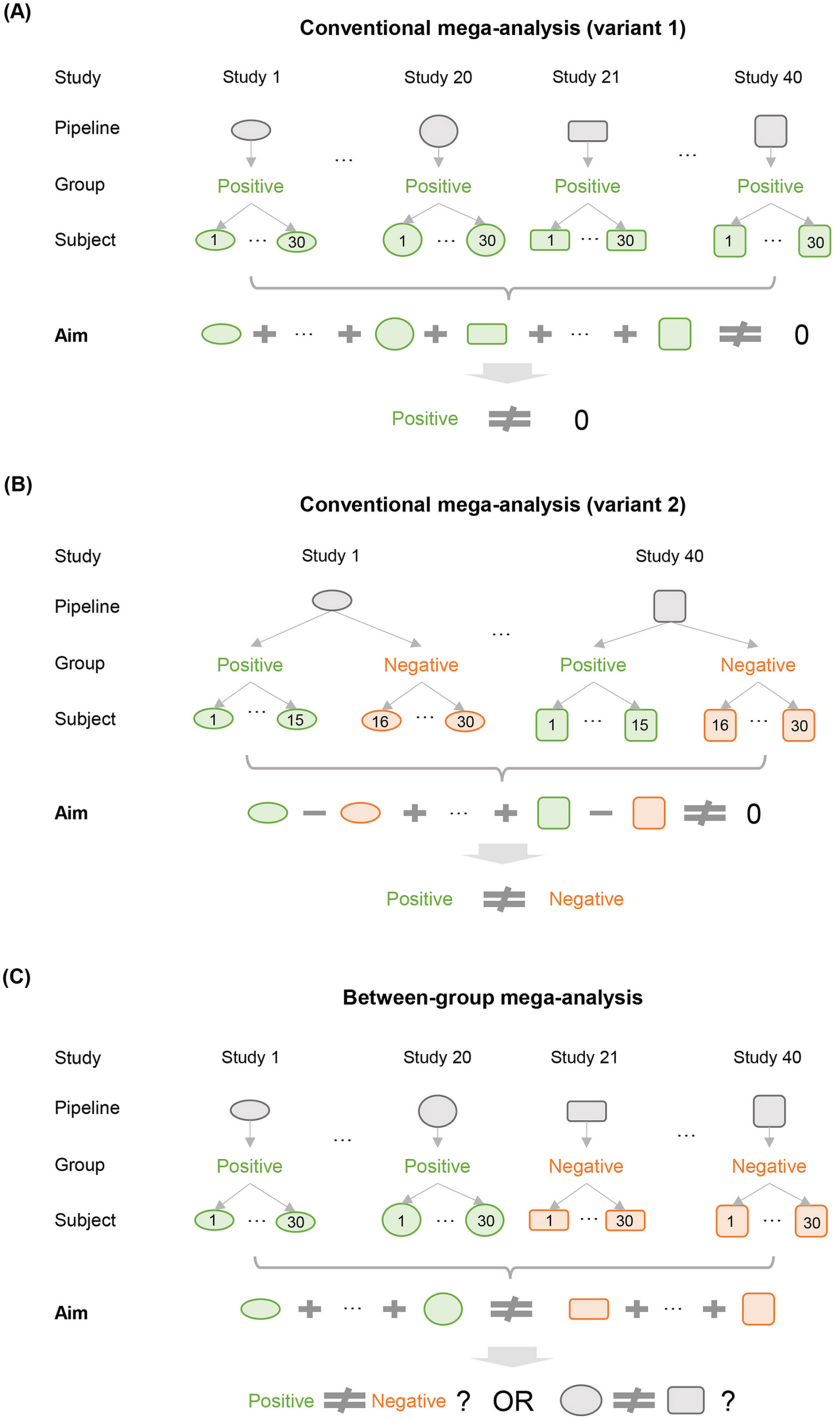
Conventional and between-group mega-analyses. (A) Conventional mega-analyses treat studies as replicates and test whether the mean of a brain feature (e.g., activation evoked by positive affective stimuli) deviates from zero across studies. As the group variable has no variance across studies, pipeline variability does not confound group effects, but improves the generalizability of findings. (B) A variant of conventional mega-analyses tests whether the mean difference between two within-study groups (e.g., individuals receiving positive or negative affective stimuli) is non-zero across studies. Since the group variable varies within studies, pipeline variability still does not confound group effects, but improves the generalizability of findings. (C) Between-group mega-analyses test whether the mean of one group in some studies (e.g., mean activation evoked by positive affect stimuli in Studies 1–20) differs from that of another group in other studies (e.g., negative affect activation in Studies 21–40). Pipeline variability confounds group effects since they covary.

Pipeline variability plays distinct roles in conventional and between-group mega-analyses. In the former, pipeline variability rarely inflates false positive rates, and instead strengthens generalizability. In the latter, it can produce spurious findings. In conventional mega-analyses, the key independent variable (e.g., positive vs. negative affect) varies within studies and the same pipeline normally applies to all subjects in the same study. Accordingly, pipeline differences do not confound the effect of interest. Indeed, between-study pipeline variability improves the generalizability of discoveries, making it desirable in conventional mega-analyses (Kiar et al., 2024; Kragel et al., 2018; Van Oudenhove et al., 2020). A generalizable finding needs to be consistently observed across multiple valid pipelines; otherwise, it could be an artifact caused by an idiosyncratic parameter choice. In this context, the strictest control over pipeline variability—analyzing data with only one pipeline for all studies—is detrimental, since one can only conclude that the finding holds under this specific pipeline. To ensure generalizability, one can introduce pipeline variability by analyzing the same data with multiple pipelines, as when conducting a same-data mega-analysis (Lefort-Besnard et al., 2025). In reality, however, it is very rare for two studies to adopt identical analytical pipelines. Such pipeline differences do come with challenges even in conventional mega-analyses. They can introduce spatial noise and bias effect size estimates (typically towards zero) if unaccounted for. To reduce bias, conventional mega-analytic studies often adjust for pipeline differences across studies with harmonization or mixed-effects models (Wang et al., 2023; Wilcox & Wang, 2023). Notably, pipeline-induced bias reflects a loss of efficiency rather than increased false discoveries.

While generalizability remains a goal, pipeline differences have a different, and more pernicious, impact on between-group mega-analyses, as discussed by Germani et al. Since different studies generally adopt different pipelines in practice, pipeline variability in this type of mega-analysis creates pipeline imbalance across groups that covaries with the variable of interest (e.g., positive vs. negative affect) at the between-study level, potentially leading to spurious findings. For example, if studies of positive affect more frequently use fMRIPrep preprocessing and studies of negative affect use AFNI, the methodological imbalance is a confounder. Unfortunately, controlling for pipeline differences in this setting also risks simultaneously removing effects of interest. If pipelines and groups are perfectly collinear (as in Germani et al.’s analyses), it becomes impossible to disentangle true positive vs. negative differences from those induced by the pipeline, and controlling for pipelines eliminates group differences altogether at the same time. As Germani et al. mentioned, between-group mega-analyses, although currently rare in the literature, may be of interest to researchers who aim to compare groups (e.g., patients and healthy participants) from different public datasets. However, the potential confounding of pipeline differences suggests that this type of analysis should be conducted with caution unless pipeline variability can be shown to have negligible influence.

Even in between-group mega-analyses, whether pipeline-induced spurious findings should be labeled “false positives” is context-dependent. A false positive occurs, by definition, if and only if (1) the null hypothesis is true (e.g., Group 1 and Group 2 do not differ), and (2) statistical tests reveal significant differences between Group 1 and Group 2. In the context of a simulation in which the null hypothesis is known to be true because the data result from a known generative model, all observed findings are, indeed, false positives. Germani et al. sample two groups of subjects at random from the Human Connectome Project multi-pipeline dataset, which ensures that all group differences are attributable to confounding. Indeed, other researchers have also described effects of confounding variables as false positives (Alfaro-Almagro et al., 2021; Wang et al., 2023). However, it is not the case that all findings of differences between groups of studies conducted in practice are false positives. Outside the simulation context, it is unknown whether the null hypothesis is true or false and therefore whether the finding is a false positive or true positive. The observed effects may well be true positive effects of methodological variables (Antonopoulos et al., 2023; Bhagwat et al., 2021; Bowring et al., 2019, 2022; Li et al., 2024; Luppi et al., 2024). For some researchers, particularly methodologists, pipeline-related differences could, indeed, be effects of interest, e.g., true differences in field strength, analysis quality, or other methodological variables. Even when pipeline differences are of no interest, in practice, they could (1) align with true effects of Group and thus boost them; (2) oppose true effects of Group and thus mask them; or (3) be independent of Group, having no effect. Thus, in the context of Germani et al.’s simulations, all group differences are false positives, whereas in actual data analyses in the field this is not necessarily the case. To avoid confusion, we recommend more descriptive terms such as “pipeline-induced spurious findings” or “pipeline-induced confounds” instead of “false positives”.

Ultimately, pipeline-induced variability may be a friend or foe, depending on the analytical goal. In typical mega-analyses testing non-zero effects across studies, methodological variability strengthens generalizability and guards against idiosyncratic pipeline-induced artifacts (Kiar et al., 2024; Kragel et al., 2018; Van Oudenhove et al., 2020). Only in the case of between-group mega-analyses does pipeline variability act as a confounder in group comparisons. Even then, pipeline-induced differences are better viewed as spurious findings rather than “false positives”. Recognizing these complexities will help understand when pipeline variability is advantageous and when it can cause problems, and more broadly recognize the contexts in which mega-analyses are trustworthy.
